# Inspiratory Muscle Training for Patients With Chronic Obstructive Pulmonary Disease: A Narrative Review

**DOI:** 10.7759/cureus.111980

**Published:** 2026-07-03

**Authors:** Bruno Bordoni, Bruno Morabito, Allan R Escher

**Affiliations:** 1 Physical Medicine and Rehabilitation, Don Carlo Gnocchi Foundation, Milan, ITA; 2 Physical Medicine and Rehabilitation, School of Osteopathic Centre for Research and Studies, Milan, ITA; 3 Oncologic Sciences, University of South Florida Morsani College of Medicine, Tampa, USA; 4 Anesthesiology/Pain Medicine, H. Lee Moffitt Cancer Center and Research Institute, Tampa, USA

**Keywords:** acute exacerbations, aecopd, chronic obstructive pulmonary disease, copd, diaphragm, guidelines, inspiratory muscle training, pulmonary rehabilitation

## Abstract

Chronic obstructive pulmonary disease (COPD) is the third leading cause of death worldwide, despite being considered a partially preventable disease. COPD includes the presence of chronic bronchitis and/or pulmonary emphysema. Treatment for these patients involves multiple approaches, including pharmacological, surgical, and rehabilitative interventions. Pulmonary rehabilitation (PR), when properly implemented, is free of harmful effects compared to medications and surgery. PR can improve symptoms, quality of life, exercise tolerance, dyspnea, and delay mortality. Inspiratory muscle training (IMT) is recommended as part of PR or as an adjunctive therapy. There is disagreement in the literature regarding the usefulness of routinely adding IMT to PR, as it does not appear to add significant clinical benefits. Looking at the literature, there is great inhomogeneity in the parameters used for IMT, and conclusions are therefore elusive. The article reviews the importance of IMT as an integral part of PR and highlights the diaphragm as more than just a respiratory muscle. A respiratory work protocol is proposed to align with current training principles.

## Introduction and background

Chronic obstructive pulmonary disease (COPD) is a respiratory disease that causes progressive airway obstruction, with structural (airway remodeling) and functional alterations in alveolar and parenchymal tissues [1]. Previous estimates and statistical projections placed COPD as the third leading cause of mortality in the world by 2030; in reality, the impact of the disease has accelerated, with it currently being the third leading cause of death [1]. In 2012, COPD was the fourth leading cause of death, with 3.1 million deaths [2]. In 2019, the Global Burden of Disease Study highlighted the deaths of 3.3 million people, making COPD the third leading cause of death in the world [3,4]. Approximately 6-7% of deaths in the world are attributable to COPD [5,6].

There are several causes that lead to the onset of COPD: cigarette smoking, chronic exposure to inhaled toxic substances, air pollution, infections, socio-environmental factors, and genetic alterations [7,8]. COPD is considered an inflammatory disease, which includes the presence of chronic bronchitis and/or pulmonary emphysema (subject to clinical confirmation and exclusion of other pathologies); the first is defined by the presence of a productive cough for at least three months during the year and for two consecutive years, while the second is defined as a lesion of the alveoli preventing gas exchange [9]. The typical symptoms reported by patients with COPD are linked to intolerance to physical activity, reduction in health-related quality of life (HRQOL), dyspnea, and dynamic/static pulmonary hyperinflation (a condition in which the lungs remain chronically or temporarily over-distended) [10]. COPD is considered a partially preventable disease [8,11].

Treatment for patients with stable COPD involves the use of pharmacology, such as bronchodilators (beta-2 agonists, short-acting beta-2 agonists (SABAs), long-acting beta-2 agonists (LABAs), antimuscarinics (short-acting antimuscarinics (SAMAs), long-acting muscarinic antagonists (LAMAs)), corticosteroids, roflumilast, theophylline, and mucolytics [12,13]. Combinations can be made depending on the patient's risk. Indications for exacerbations may vary, such as the use of macrolides or LABAs/corticosteroids/LAMAs [12-14]. Some precautions should be combined with the usual medications, such as advice to improve lifestyle habits (e.g., not smoking), to follow influenza and pneumococcal vaccinations, and to improve caloric intake where necessary [13].

In patients with severe COPD, surgical options include transplantation, volume reduction surgery, pulmonary bullectomy, and bronchoscopic approaches [13,14]. The goal is to improve symptoms, HRQOL, exercise tolerance, dyspnea, and delay mortality.

Another treatment for COPD with the same goals is pulmonary rehabilitation (PR). The latter is defined as: “a comprehensive intervention based on a thorough patient assessment followed by patient-tailored therapies that include, but are not limited to, exercise training, education, and behavior change, designed to improve the physical and psychological condition of people with CRD (chronic respiratory disease) and to promote long-term adherence to health-enhancing behaviors” [15,16]. PR is managed by an interprofessional team, where the patient is educated on healthier behaviors and functional independence; following a PR program allows for many benefits, such as reducing dyspnea, increasing exercise tolerance, improving HRQOL and psychological well-being, and reducing the number of readmissions and the mortality rate after discharge [5,14,16].

Although guidelines encourage the use of PR for COPD patients (strong recommendations with moderate-quality evidence), only 1.9-5% of patients access PR [14,17-20]; PR shows poor adherence. There are various reasons for this: lack of suitable facilities, excessive distance and lack of transportation, socioeconomic and racial disparities depending on the geopolitical area, and comorbidities [16,17].

The foundation of PR is based on physical activity [5]. The recommendations that highlight the importance of exercise lack precise indications on how to manage training volumes, just as there are no indications on how to adapt PR based on the patient's comorbidity or endotype/phenotype [9,16,18,19]. Classifying COPD by endotype and phenotype allows us to move beyond the concept of a "single disease," guiding physicians towards targeted and personalised treatment. Phenotypes describe how the disease manifests, while endotypes explain the underlying biological mechanism. In fact, not all COPD patients obtain the same benefits from PR [10].

Another important element that is not adequately taken into consideration is diaphragm muscle training, which is included in a generic context as inspiratory muscle training (IMT), both in Western and Asian and Eastern countries, regardless of the stability or exacerbation of COPD patients, and in the maintenance phase [9,16,17,19-21]. Not only is the diaphragm the motor muscle of respiration, but its contractile capacity is an independent indicator of survival in patients with severe COPD and a parameter to identify the type of patient who will respond best to PR [10,22].

The article reviews the importance of IMT as an integral part of PR, evaluates the significance of the diaphragm as more than just a respiratory muscle, and proposes a respiratory work protocol that better reflects our current understanding of training principles. The final conclusions of the narrative review suggest using IMT throughout the patient's life, and not just as a moment inserted into the PR process.

## Review

Research methods/strategy

This narrative review prioritized English-language articles published in PubMed with clinically oriented evidence and rehabilitation implications, highlighting the most recent information on the topic of the article. The review included narrative and systematic reviews, observational studies, and relevant guidelines, using combinations of the following terms and keywords: chronic obstructive pulmonary disease; COPD; diaphragm; pulmonary rehabilitation; inspiratory muscle training; guidelines; acute exacerbations; AECOPD. Additional relevant literature was derived from reading the articles included in this narrative review.

Benefits of PR in stable COPD

PR is recommended for patients with stable COPD and during exacerbations. While its organization varies from country to country, PR consistently enhances health-related quality of life (HRQOL), boosts physical work capacity under stress, lessens feelings of breathlessness, reduces fat mass in those with obesity, and improves lung function along with respiratory muscle strength [23]. For adults with stable COPD, the American Thoracic Society (ATS) published a recent review, analyzing 82 trials with 4,674 patients, confirming the benefits that PR can provide. This review showed a significant clinical increase in functional capacity (improvement of the six-minute walking test (6MWD), the incremental shuttle walk test, and peak work rate parameters) and improved parameters related to the assessment of dyspnea (Chronic Respiratory Disease Questionnaire-dyspnea, transitional dyspnea index) [18]. HRQOL parameters improve with significant clinical improvement (St. George’s Respiratory Questionnaire, Chronic Respiratory Disease Questionnaire-fatigue/emotion/mastery) [18]. The ATS review highlighted a lack of clinical statistical significance regarding the mortality rate among COPD patients who underwent PR, compared to COPD patients who did not undergo rehabilitation (reviewing 42 trials) [18]. The ATS recommendations for stable COPD patients to undergo PR are strong but with moderate-quality evidence [16,18].

According to the British Thoracic Society (BTS), the benefits of following PR for patients with stable COPD are greater than those of bronchodilator drug therapy; it improves HRQOL, exercise tolerance, and dyspnea. According to BTS, there is a strong evidence base for following PR for patients with stable COPD [17].

According to the Global Initiative for Chronic Obstructive Lung Disease (GOLD), founded in 1998, PR in stable patients or those at risk of exacerbation improves the sensation of dyspnea, the ability to perform exercise with less fatigue, and improved functional and psychological status (evidence A); PR reduces hospitalization in patients who have recently experienced an exacerbation (evidence B). As with BTS, GOLD highlights (evidence A) that the lack of physical activity in patients with COPD is directly related to the mortality rate [24].

The Japanese Respiratory Society (JRS) indicates PR as strongly recommended (evidence A); PR improves the ability to perform physical activity with less fatigue, increases HRQOL values, and decreases the mortality rate [25,26].

The Internal Medicine Professional Committee of the World Federation of Chinese Medicine Societies recently published recommendations on PR in conjunction with traditional Chinese medicine. This committee recognizes the benefits of PR, with improvements in HRQOL, exercise tolerance, and pulmonary function, a reduction in disability and mortality rates, as well as a decrease in the rate of re-hospitalizations. As for recommendations in pursuing PR and traditional Chinese medicine, the evidence is strong but of low quality [27].

The Saudi Thoracic Society (STS) agrees in recognizing the benefits of PR in stable COPD patients. It improves functional and psychological status, decreases symptoms such as dyspnea and fatigue, and improves HRQOL parameters [28]. The STS states that patients with FEV1 between 50% and 80% of the predicted value, and patients with an FEV1 reaching 50% of the predicted value, can follow PR (evidence B).

In line with previous guidelines, the European Respiratory Society (ERS) considers PR for stable COPD as “undisputed” [29].

Take-home message: In stable patients, major guidelines undoubtedly recommend performing PR.

Benefits of PR with exacerbation

Exacerbation is defined as a worsening of symptoms within two weeks [24]. COPD patients with an acute exacerbation (AECOPD) are at increased risk of rehospitalization and mortality after hospital discharge, according to ATS estimates [16]. The ATS recommends pursuing PR in this clinical setting only after approximately four weeks from discharge (and thus returning to stable status). The benefits of PR after exacerbation demonstrate a reduction in hospital readmissions, an improvement in parameters related to 6MWD, HRQOL, and decreased dyspnea, but not a decrease in the mortality rate. This latter parameter is derived from the analysis of nine trials with 995 patients [16]. PR after an exacerbation event has a strong recommendation with moderate-quality evidence [16,18].

According to the BTS guidelines, PR should be performed after hospital discharge (one month later), that is, when the patient is stable. The benefits of PR mirror the ATS data [17].

GOLD recommends using drugs (bronchodilators - evidence C, corticosteroids - evidence A, antibiotics - evidence B) and instrumental approaches in the acute phase (non-mechanical ventilation - evidence A), but not PR [24]. GOLD recommends following PR only after discharge and when the patient is stable. During AECOPD, females demonstrate greater acuteness, with greater dyspnea and cough, and a slightly lower FEV1 value than males [11].

The ERS guidelines are in line with other recommendations, advising to start PR only three weeks after discharge from the acute phase [29]. ERS emphasizes that performing PR during the acute phase could be detrimental for the patient (conditional recommendation and low quality of evidence, 2017) [30]. A recent review evaluated two meta-analyses of trials (between 2020 and 2022), overturning the ERS statement of 2017, where patients in the acute phase who followed PR during hospitalization improved some functional and quality of life parameters, dyspnea, and reduced hospitalizations for exacerbations [14].

The Internal Medicine Professional Committee of the World Federation of Chinese Medicine Societies, regarding PR in patients with AECOPD, does not recommend physical activity during hospitalization, but only pharmacological and instrumental approaches [27]. The STS recommends the use of PR for hospitalized patients, with type A evidence [28].

It should be noted that symptom relief after an exacerbation occurs after 7-10 days; 20% of the patient population presents significant unresolved symptoms after two months [31].

Major guidelines from European and American countries (but not Asian guidelines) strongly recommend performing PR for hospitalized patients.

Additional benefits of PR

We know that PR (through aerobic and anaerobic activity) aims to alleviate symptoms, improve physical activity capacity, provide prevention and education, and attempt to reduce mortality [32]. We know that it helps patients in many ways, but it is consistently underutilized [32].

PR can mitigate the inflammatory response by decreasing the level of adipokines (chemerin), which have the functional characteristic of being chemoattractants for natural killer and plasmacytoid dendritic cells, and macrophages [19,33]. The level of chemerin increases during exacerbation, with a parallel relationship between inflammatory values (C-reactive protein, interleukin 8), and the frequency of hospitalization; chemerin decreases when the acute event declines [33]. The precise mechanisms of action of chemerin and COPD are not yet fully clarified. Physical activity for cancer patients within PR reduces the levels of signal transducer and activator of transcription 3 (STAT3), a protein and transcription factor [34]. This particular phenomenon was noted in animal models that included aerobic activity, probably by inhibiting the activity of the protein and reducing leukocyte activation [35]. This particular phenomenon was noted in animal models that included aerobic activity, probably by inhibiting the activity of the protein and reducing leukocyte activation [35]. Another study on an animal model (a group of smokers genetically modified to have COPD), which was subjected to aerobic activity, highlighted the decrease of inflammatory processes, probably by inhibiting Wnt/β-catenin-peroxisome proliferator-activated receptor (PPAR) γ signaling [36]. In a human COPD model, physical activity (aerobic and anaerobic) reduces the inflammatory levels of several biomarkers, such as pro-inflammatory cytokines, levels of leukocytes, lymphocytes, and monocytes, C-reactive protein (CRP), and chemoattractants [19].

COPD patients experience muscle mitochondrial dysfunction and oxidative damage, impairing motor function; aerobic and anaerobic exercise reduces this damage [37]. One reason could be related to the levels of irisin, a myokine secreted by moving muscles; the higher the levels, the better the amount of physical activity performed, as it protects against oxidation [38]. Oxidation and activation of nicotinamide adenine dinucleotide phosphate oxidase stimulate ferroptosis, programmed cell death [39]. Irisin probably defends cells from oxidation through the stimulation of nuclear factor erythroid 2-related factor 2 (Nrf2), with the subsequent stimulation of the production of antioxidant substances [19,40].

PR improves muscle fiber functionality. In an animal model (aged mouse model), strength training stimulates mTORC1 (mammalian target of rapamycin complex 1, a protein complex that allows protein synthesis), HIF-1α (hypoxia-inducible factor, a protein that improves oxygen delivery to the cell), and mTORC1-AMPK (5' adenosine monophosphate-activated protein kinase, an enzyme essential for physiological metabolism in the cell) pathways [41]. These biochemical responses reduce the levels of inflammatory biomarkers (interleukin-1beta, tumor necrosis factor alpha), increase the levels of interleukin-6 (a metabolic sensor that allows maintaining adequate cellular energy levels), interleukin-10 (a cytokine with anti-inflammatory properties), and interleukin-15 (an inflammatory cytokine that improves protein synthesis) [19,42-44]. Resistance training (anaerobic activity) reduces the levels of myostatin (a protein that inhibits protein hypertrophy), increases the levels of follistatin (a glycoprotein that regulates the efficiency of cellular differentiation), increases the levels of FNDC5 (fibronectin type-II domain-containing 5, involved in the phenotypic regulation of fibers) and IGF-1 (insulin-like growth factor-1, a muscle hormone essential for anabolic processes); increases the levels of semaphorin 3 (a chemorepellent axonal guidance protein that intervenes in the management of the synaptic junction) [19,45-48].

Moderate intensity (endurance-aerobic) training stimulates the synthesis of the protein PGC-1alpha (peroxisome proliferator-activated receptor gamma coactivator 1-alpha, essential for mitochondrial biogenesis), which improves ATP utilization and positively influences the metabolic pathway for antioxidant defense via the Keap1-Nrf2-ARE pathway (Keap1 or Kelch-like ECH-Associating protein 1, ARE or antioxidant response element) [19,49]. Endurance training improves vascular function, where the extent of endothelial dysfunction is related to low FEV1 values [50]. Endothelial dysfunction is common in COPD patients, with stiffer, atherosclerotic, and calcified arteries and a lower circulation of endothelial progenitor cells; this leads to a high incidence of cardiovascular disease [50,51]. Training reduces fibrinogen levels and vessel contraction capacity (reactive hyperemia index) in patients with COPD, probably due to increases in nitric oxide [19,51].

Endurance training raises the lactate threshold, increasing exercise tolerance; this adaptation allows for a reduction in dyspnea during physical activity [19]. This event could explain the decline in inflammatory blood values. Elevated blood lactate values in COPD patients result from peripheral muscles stressed during physical and daily activity; the muscles undergo structural (myopathy), functional, and phenotypic alterations (increase in white fibers), a decrease in capillarization, and oxygen diffusion [52,53]. Lactate undergoes lactylation, a post-translational modification of proteins where histones (protein molecules related to DNA) are altered, creating a covalence between the lysine residues and the lactyl group [54]. This covalence influences the macrophage response (M1) and the immuno-inflammatory responses, with an elevation of lymphocytes and pro-inflammatory cytokines (such as interleukin-17). This biochemical mechanism promotes the maintenance of a chronic inflammatory environment and tissue damage [55]. These biochemical mechanisms can cause damage to lung structures in COPD patients, as shown in animal models [56].

Despite the reduction in inflammatory levels after PR in patients with COPD, there does not appear to be a structural and functional recovery adaptation at the pulmonary and alveolar levels [57].

**Figure 1:**
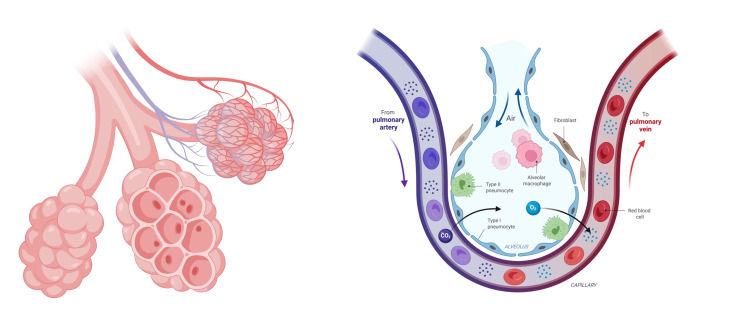
The figure schematically illustrates the complexity of healthy alveoli and blood vessel coverage (right), and the gas exchange between an alveolus and the blood system (left). These mechanisms are chronically altered in the presence of COPD. Image credit: Created by Bordoni B. via a subscription on BioRender.com

Take-home message: Performing PR helps lower levels of systemic inflammation and oxidation (Figure 1).

The diaphragm and COPD

In patients with COPD, transdiaphragmatic pressure (Pdi) derived from the diaphragm is approximately 40% lower than in healthy subjects; the muscle fibers appear shorter, likely due to the chronic inspiratory posture of these patients [22,58].

In this context of chronicity (inspiratory attitude), the respiratory accessory muscles (scalene, sternocleidomastoid, pectoralis major/minor, latissimus dorsi, erector spinae, trapezius) become less elastic and have difficulty compensating for the diaphragm dysfunction [6,10,58]. The diaphragm in a lower position than normal will have less apposition area (and further less strength), and a lower capacity to maintain adequate tension. Patients with COPD have a diaphragmatic contractile strength 35% lower than that of healthy subjects [59]. A recent study compared the images of the diaphragm movement through computed tomography and magnetic resonance images in patients with COPD [60]. The analysis carried out highlighted an asynchronous movement of the hemidiaphragm, with a greater lack of coordination in patients classified as GOLD-IV and in the posterior portion; normally, it is the posterior portion that moves more. This asynchrony determines a greater inability to inspire [60,61]. The phrenic nerve undergoes pathological adaptations with functional alterations (neuropathy with myelin damage); the greater the phrenic neurological damage (in particular, the left nerve), the greater the presence of hyperinflation [58]. The maximum excursion of the diaphragm (DEmax) during a respiratory act is reduced [10]. These pathophysiological adaptations are found at every stage of the disease [22].

From an ultrastructural point of view, the fibers undergo many alterations. The percentage of aerobic or type I fibers increases, to the detriment of anaerobic or type II fibers, even though the phenotypically increased fibers are poorly functional [58]. The fibers show signs of atrophy, fibrosis, and myolysis; the fiber's motor protein, the myosin heavy chain (MHC), is reduced in quantity (approximately 30-50%) [58]. There is an accumulation of intracellular calcium (Ca2+) due to dysfunction of the sarcoplasmic reticulum, which not only stimulates mechanisms related to apoptosis but also binds to inorganic phosphorus (Pi), inhibiting the sensitivity of troponin C and decreasing the formation of actomyosin bridges [62]. The diaphragm fiber is weaker and more subject to microtrauma. The proteins nebulin and titin undergo a reduction and a decline in elasticity, respectively, in patients with COPD, making the fibers less compliant to the present tension [58,63]. The sarcomere tends to lose its alignment, the serial number, with the accumulation of proteins in the Z areas; collagen tissue increases within the space left by the reduction of sarcomeres, with a shortening of approximately 28% compared to healthy subjects [58].

Many inflammatory substances are present within the diaphragm, such as tumor necrosis factor-alpha, interleukin-1 and 8, as well as CRP; this triggers biochemical reactions that tend to destroy and remodel the fibers, such as the non-canonical NF-κB pathway, canonical NF-κB pathway, and the myostatin-mothers against decapentaplegic homolog 3 (Smad3) pathway [22,58]. Systemic inflammatory levels are directly related to the severity of COPD [22]. We find in the diaphragm phenomena related to oxidation, which lead to apoptosis and proteolysis [22].

In this non-physiological environment, diaphragm fibers are less able to use cellular repair mechanisms (increased myostatin levels) and have reduced satellite cell chemotaxis [58]. It seems that the capillary scaffolding of the fibers is preserved, while we do not know how the lymphatic system adapts in COPD patients [58].

Take-home message: COPD alters the structure and function of the diaphragm muscle, changing its shape (inspiratory position) and function (less force is expressed).

Central adaptations of the respiratory centres

The persistence of the disease can lead to cognitive dysfunction, functional alterations (less activity compared to healthy subjects) of the anterior insular cortex (proprioceptive dysfunctions, part activated by inspiration); general cortical alterations (loss of gray matter) are shown to be positively correlated with forced vital capacity (FVC) (in particular with the medial orbitofrontal area, frontal pole and middle temporal area), and negatively correlated with FEV1 (in particular, with the frontal pole area, part activated by inspiration) [64-66]. Some authors speak of a "lung-brain axis," meaning a biunivocal relationship between respiratory problems and the brain; for example, the pulmonary microbiome has been shown to alter the immune responses of the central nervous system [67].

Although approximately 60% of COPD patients may experience cognitive decline, we do not have a comprehensive explanation for this phenomenon [68]. Hypoxemia and hypercapnia are likely to reduce levels of neurotransmitters such as dopamine and serotonin; furthermore, the same pro-inflammatory cytokines present in the blood circulation are also present in the brain, triggering neuroinflammation [68].

What do we know about the central respiratory pattern generator (CRPG) and the adaptations it undergoes in the presence of COPD? In the presence of hypoxia and chronic respiratory problems, such as in COPD, the nucleus of the solitary tract (the vagal nerve centre for respiratory rhythm) undergoes non-physiological adaptations with an altered relationship with the sympathetic system; this leads to long-term facilitation of the phrenic nerve [69,70]. We have no information on what happens to the CRPG in the presence of COPD, which is paradoxical, as these are the brain areas that determine and specify the rhythm and quality of breathing. These regions enable the diaphragm and accessory muscles to facilitate the intake of air into the lungs. The lungs without the diaphragm (or instrumentation that takes the place of the diaphragm) cannot insufflate. The contraction and relaxation of the diaphragm are responsible for 80% of the overall movements during the eupneic act [71]. Diaphragm weakness decreases ventilatory capacity and the ability to perform physical activity [6].

Take-home message: COPD alters the structure of some brain areas and stimulates the development of cognitive decline.

Diaphragmatic benefits with PR/IMT

Inspiratory muscle training (IMT) is considered a specific training approach for the inspiratory muscles, and not necessarily for the diaphragm [6]. And it is not necessarily always included in PR [20]. By applying variable resistance to a specific instrument placed during inspiration, the inspiratory muscles can increase aerobic and/or anaerobic performance [6,22]. A recent review evaluating 59 studies with a total of 2,467 patients highlighted that adding IMT to a PR program is unnecessary, even though IMT without other physical activity improves patients' overall performance [9]. Despite this known data, the ATS/ERS recommends integrating IMT into PR, particularly when the patient exhibits severe respiratory weakness [72].

The reasons are unknown. Furthermore, we do not know what happens to the diaphragm after IMT, from an ultrastructural, metabolic, and electric (CRPG/phrenic) point of view. IMT after 8 weeks at home decreases diaphragmatic intervention with increasing effort, thereby better regulating the myoelectric spectrum (neural respiratory drive) [73]. At the end of 12 weeks of usual PR (without IMT), if the patient continues for another 12 weeks with an IMT-only setting, increases in DEmax, maximum inspiratory force (PImax), oxygen use at peak effort (peak Vo2), and a decrease in dyspnea are recorded (38 patients with stable COPD), compared to patients who did not follow IMT after PR [72]. The mechanism by which dyspnea improves is unknown, and the precise neural pathways underlying this phenomenon are not well characterized; for some authors, it is a "cognitive illusion" [72,74,75]. IMT can increase transdiaphragmatic pressure (Pdi) [76].

One study compared PR and IMT in AECOPD (experimental group of 8 patients) versus usual PR (8 patients) [76]. In the experimental group, they measured better values related to exercise tolerance (6MWD), HRQOL, PImax, and a more homogeneous myoelectric activation of the diaphragm and sternocleidomastoid (less electrical activity with greater effort) [77].

We know that after IMT, the external intercostal muscles undergo a redistribution of fiber phenotype and an increase in the volume of anaerobic fibers [9].

Take-home message: IMT in patients with stable COPD improves DEmax, PImax, peak VO2, and Pdi.

Why IMT? The diaphragm is not only involved with the lungs

Besides the fact that the diaphragm is the primary muscle for inhalation and for drawing air into the lungs, there are other considerations.

When the diaphragm is functioning properly, the lungs send afferents to the CRPG (stretch receptors) via the vagus nerve, stimulating the Hering-Breuer reflex, which helps prevent hyperinflation and the formation of atelectasis. Likewise, diaphragmatic receptors (mechanoreceptors), when stimulated by breathing, send physiological afferents to the CRPG to properly manage the function of the phrenic nerve [74]. The balance between the sympathetic and parasympathetic systems also regulates the rhythm of breathing. Other receptors, the chemoreceptors located in the carotid sinus, send afferents to the CRPG via the glossopharyngeal nerve with each breath [74]. Similarly, the aortic depressor nerve (aortic nerve, a branch of the vagus nerve) sends afferents from the aortic arch to the CRPG with each breath [78]. In fact, each breath activates all the body's receptors (interoception), stimulating not only the respiratory centres but also many cortical and subcortical areas. This allows breathing to adapt to the body's needs in each situation [61].

We know that COPD alters the structure and function of the diaphragm and lungs, thereby impairing afferent signaling and the ability of higher centres to adequately process the information they receive regularly, regardless of the stage of the disease. Chronic receptor alteration leads to systemic changes. Bodily movement and cognitive aspects necessarily change [7,59,73].

The diaphragm should be considered not only for its respiratory function, but also for other functions for which it is essential to the patient's health, and to protect them from the negative consequences of respiratory dysfunction.

Falls, pain, anxiety, and depression

Patients with COPD have a higher rate of falls (30-55%) than non-COPD patients (1.17 to 1.49 falls/person-year), resulting in imbalance and gait problems [79-81]. There is a close link between COPD and falls, and the ATS/ERS recommends including the patient's balance ability in routine assessments [82]. The reasons are varied: proprioceptive impairment, sarcopenia (which worsens with exacerbations), chronic pain, atrial fibrillation, malnutrition, anxiety, depression, and cognitive impairments [59,83-87].

There appears to be a relationship between respiratory muscle weakness and proprioceptive impairment in patients with COPD [88]. There is a direct relationship between decreased postural control and diaphragmatic impairment in patients with COPD, though this relationship is not necessarily related to respiratory action [89].

In non-COPD subjects, diaphragmatic weakness is associated with increased pain episodes linked to proprioceptive alterations and loss of correct postural control [90,91]. Further studies will be needed to confirm these hypotheses. Furthermore, there seems to be a relationship between the presence of chronic pain (66-95% in patients with COPD), anxiety and depression (up to 80% in COPD patients), and dyspnea (70-82% of patients with COPD) [74,83,92-97]. Proprioceptive alteration, anxiety and depression, chronic pain, and dyspnea increase the mortality and exacerbation rate [83,92-97].

A common thread may link these symptoms in COPD patients with structural and functional alterations of the diaphragm [74]. Breathing begins through the nose (pre-inspiration phase), stimulating the visual cortical areas, the limbic areas, and the primary sensory cortex (part of the proprioceptive system), and triggering various neural oscillations that influence cognitive and emotional processes [98,99]. Further studies will be needed to confirm these hypotheses. COPD patients may present with a high prevalence of nasal respiratory problems, such as chronic rhinosinusitis (15.3-82%) [100,101]. We can only assume that, if the strength and range of movement of the diaphragm are decreased, possible proprioceptive disturbances and emotional/motor alterations may arise from nose-diaphragm problems. In COPD patients, the amygdala (limbic area) is activated early, before the onset of dyspnea [74]. Furthermore, the limbic area (the same amygdala) processes nociceptive information; this pre-inspiration phase activates the prefrontal cortex, which is involved in processing nociceptive information. The human body does not distinguish between emotions, pain, and movement [102]. We could hypothesise using these relationships to further recommend the IMT approach within PR, noting that closed nasal passages can further fatigue the diaphragm, which will encounter greater resistance during inspiration.

A second phase of pre-inspiration involves the upper airways, namely the lingual complex. CRPG activates the lingual complex to open the upper airways, allowing air to pass into the bronchi; the tongue pushes the hyoid bone forward during inhalation [61,102]. All the nerve pathways involved in controlling the movement of the laryngeal area, such as the trigeminal nerve, the facial nerve, the glossopharyngeal nerve, the vagus nerve, and the hypoglossal nerve, are activated; this activation will, in turn, stimulate different cortical and subcortical areas. Areas of the motor cortex, the limbic area, the cerebellum, and the putamen are activated; these areas will prepare the body for movement by managing the proprioceptive information that arrives and will arrive, and which will be managed as pain, movement, and emotions [61,102-104]. One of the comorbidities in COPD patients is dysphagia (59.01%), which results from incorrect central coordination between swallowing and breathing; patients experience a prolonged expiratory phase during swallowing (solid and liquid) [105,106]. Dysphagia increases morbidity and mortality rates, as do anxiety, depression, and stress [107]. In stroke patients, there is a direct relationship between diaphragmatic weakness and the presence of dysphagia [108,109]. In stroke patients, training to improve diaphragmatic function decreases the occurrence of accidental aspiration due to dysphagia [110]. Further studies will be needed to confirm these hypotheses. There is a direct relationship between diaphragmatic behavior and swallowing mechanisms [111].

Another problem related to lingual behavior and COPD is the presence of snoring/sleep apnea, defined as overlap syndrome (OS), with a reported incidence of 19-84% [112,113]. In patients with obstructive sleep apnea (OSA), the tongue shows reduced stereognostic (and proprioceptive) capacity, with altered electrical function and incorrect coordination of the temporal activation of the diaphragm and the tongue [114]. The same dysfunctional lung sends aberrant afferents from the slow-conducting, unmyelinated interstitial receptors (J receptors, or juxtacapillary receptors, or pulmonary C-fiber receptors) to the XII cranial nerve; this chronic pathological mechanism could create a dysfunctional environment among the tongue, lung, and diaphragm [115]. Another line of reflection in patients with COPD is the evaluation of the relationship between the tongue and the diaphragm, and whether it could be included as a motivation to perform IMT within PR. We recall that chronic rhinosinusitis has a close relationship with the presence of OSA (and with obesity, a condition commonly found in COPD and diaphragmatic weakness); therefore, constituting a recognizable pattern involving the same interdependent airways [116-118].

The inspiratory phase involves diaphragmatic movement. Pressure changes between the thorax and abdomen stimulate many receptors, with about 95% sending signals to the nucleus of the solitary tract via the spino-solitary pathway and around 5% to the spinal trigeminal nucleus via the spino-trigeminal pathway [99]. All afferent information concerns proprioception. From the spinal trigeminal nucleus, further steps involve the Gasser ganglion, which sends afferents back to the NTS. The proprioceptive information that reaches the NTS is exchanged in a biunivocal manner with the cerebellum and the vestibular system; these two areas process and manage information related to pain, emotions, and movement [99]. The NTS transmits processed information to cortical and subcortical regions, including the limbic, motor, and sensory areas, for further processing. The processed information becomes higher-order and returns to the NTS, which sends inhibitory efferents towards the rostral ventrolateral area of the spinal cord to slow down the activity of the pre-neurons of the sympathetic system [99]. This mechanism improves the electromyographic spectrum (greater strength expressed by skeletal muscles), enhances cognitive and emotional status, and raises the pain threshold. Each breath allows the body to use its motor, emotional, and cognitive resources at their best [99]. A pathological adaptation of the diaphragm, as found in patients with COPD, could be a strong cause of emotional and movement alterations and the persistence of chronic pain [93,95].

The diaphragm tends to move faster than in healthy subjects; this would lead to an early onset of fatigue [73]. The DEmax is reduced in COPD due to biomechanical disadvantages and myoelectrical and structural alterations, forcing other respiratory accessory muscle areas to intervene, causing competition with the diaphragm for adequate vascularization [73]. Furthermore, if the accessory muscle areas intervene more to help the patient in breathing, they will be weaker in performing motor tasks [10]. The same thing happens to the diaphragm. The diaphragm must contract whenever movement of the limbs and trunk is involved, or there is a need to maintain movement; one third of its contractile force is used for posture and movement [58,59,61,99]. Diaphragmatic contraction allows for the creation of an adequate intra-abdominal pressure (IAP), which stabilizes the dorso-lumbar area and stimulates proprioceptive receptor activation for the neuromotor responses mentioned above [99]. In COPD, it is necessary to evaluate DEmax to assess the patient's motor capacity preventively (through transesophageal diaphragm electromyography or a simple ultrasound examination) [59]. DEmax is more important than FEV1 and PImax in predicting the effectiveness of PR in COPD patients [119]. The same concept can be used in patients with COPD/OSA [120].

Higher DEmax means greater functional achievements (6MWD) [119]. We could hypothesize that the greater the diaphragm excursion, the greater the neuromotor and postural capacity will be, thanks to a greater solicitation of the proprioceptors and a greater capacity to produce IAP. IMT (and endurance training) demonstrates a clinically relevant improvement in balance ability in patients with COPD; this adaptation should reduce the percentage of falls [121]. With IMT, the myoelectric spectrum of intervention of the diaphragm is reduced [73]. This could mean that the diaphragm is involved to a lesser extent in creating a correct posture and more coordinated movements, intervening better in the act of breathing. An improvement in functional capacity improves pain tolerance in COPD [122]. IMT can improve cognitive function (memory and attention) in patients with COPD (and COPD/obese) [8,123]. Improving respiratory function in patients with COPD helps to reduce the state of anxiety [124,125]. These COPD studies, to the best of the authors' knowledge, have never considered the relationship between the movement of the diaphragm and the proprioceptive responses that stimulate the cortical and subcortical areas involved in the emotional and cognitive aspects, as well as the nociceptive aspect.

Take-home message: A diaphragm with altered structure and function in patients with COPD could be a source of non-respiratory disorders, such as an increased incidence of falls, the presence of anxiety and depression, and a lowering of the pain threshold.

COPD and the cardiovascular system

The systemic nature of COPD negatively affects the cardiovascular system, regardless of the patient's phenotype, with a percentage that can reach 70% [126,127]. The reasons are multiple and/or concomitant. Some risk factors may coincide, such as smoking (increases arterial stiffness), air pollution (PM2.5 induces systemic inflammation and endothelial alteration) [127,128]. Hypoxemia and alteration of the vascular structure of the lungs can contribute to right ventricular dysfunction; hyperinflation reduces left ventricular filling and induces an increase in vascular resistance in the lungs, creating a vicious circle [127]. These cardiovascular events (myocardial infarction, heart failure) increase the rate of mortality and exacerbation, compared to people without COPD; there is a 2.98-fold increased risk of developing cardiac problems, with a 20% higher mortality rate in AECOPD patients [127,129]. Another shared risk factor is visceral fat and air pollution, where exposure to PM2.5 (and NO2) increases fat accumulation; increased visceral fat increases the risk of cardiovascular events in patients with COPD. The pathophysiological difference between COPD and cardiovascular events is very subtle [130,131].

One of the cardiovascular risk factors in patients with COPD (to the best of the authors' knowledge), which is rarely considered clinically, is morpho-functional diaphragmatic dysfunction. Inspiration allows venous return and the creation of adequate right atrial pressure gradients and the correct management of right cardiac chamber pressures, through the balance between the preload tension generated by inspiration (stretch) and right stroke volume [132]. Simultaneously, inhalation increases left ventricular afterload and aortic diastolic pressure gradients [132]. Expiration fills the left cardiac chambers, reducing aortic diastolic pressure gradients and allowing for correct left ventricular stroke volume. Myocardial work is thus influenced by the contractile capacity of the diaphragm [132]. This respiratory-cardiac mechanism allows for physiological circulation, exhaustive central and peripheral pressures, and a balanced autonomic response. If this relationship is disrupted, due to diaphragmatic dysfunction, the result will most likely be the onset of cardiac pathologies [132].

A functioning diaphragm is so important for a weakened heart (chronic heart failure) that one of the therapies available to the patient is the implantation of a device for electrical stimulation of the diaphragm (synchronized diaphragmatic stimulation, SDS). SDS accommodates intrathoracic pressures in synchrony with the cardiac cycle [133]. We can affirm that functional, morphological, metabolic, and neurological alterations of the diaphragm affect intrathoracic pressures and, over time, can induce pathological cardiac and pulmonary adaptations in patients with COPD.

Take-home message: The diaphragm plays an important role in cardiovascular function and health; in patients with COPD, the cardiovascular system is altered, likely related to diaphragm dysfunction and structural remodeling.

Literature and parameters for IMT

Across the various experimental studies and related reviews, there is no single approach to organizing IMT, regardless of COPD and AECOPD.

The most recent Cochrane review on IMT (2023) evaluated 55 randomized controlled trials (RCTs), comparing the presence or absence of IMT within PR, and with stable COPD patients [9]. The inferred results indicate that IMT has a weak effect on the perception of dyspnea, and that functional improvement and quality of life are observed both when IMT is present in the PR process and when it is performed as an exclusive activity. Moreover, the workload differences are striking. IMT was introduced in 1976 for patients with asthma and COPD, and since that time, a shared line has not yet been found [9]. The concept is to increase the resistance to be overcome during inspiration through various aids available on the market (none better than another); the resistance, when possible, must increase gradually to create a more effective adaptation in strength and resistance, improving the quality and quantity of inspiratory flow [9]. The applied resistance ranges from a minimum of 9% of PImax to 133%; the days on which IMT is performed range from two days a week to every day, with a minimum of two weeks to 24 weeks. An IMT session typically lasts 5-60 minutes, involves 10-36 resisted breaths in 2-15 sets, and intensity is increased by 5-30% over the initial load at variable intervals. The pauses between one set and the next, described only in rare cases, vary from 5 seconds to 10 minutes, for 4-5 minutes of duration for each set. Often, studies have been conducted at home for IMT, with varying levels of supervision [9].

Some studies did not vary the training load, while other studies did not specify the details. How to insert IMT with PR is not specified, with respect to the intensity and type of metabolism used in the training. Training is not expected to be sinusoidal, but always in a straight line; training always has the goal of increasing the resistance to be overcome (straight line), and a decreased training load is never expected to allow for correct recovery times, avoiding overtraining syndrome (persistent decline in performance, chronic fatigue, depression, and stress) [134].

Only one study specified the metabolic typology for IMT, aerobic (60% of PImax) and anaerobic (80% of PImax); aerobic for red fibers and anaerobic for white fibers [9].

More recent studies used IMT at home for 12 weeks, with stable COPD, after a "standardized" PR of 12 weeks; the protocol consisted of 30 breaths for each set, for two sets, starting from a PImax of 30% until reaching a resistance of 50% of the PImax [72]. Another study compared IMT in one group with diaphragmatic electrostimulation in another: patients used a device to vary the pressures to be overcome for 30 breaths, 3 sessions per week, over 8 weeks. There were no variables such as the pressures used for the measurement of PImax, the duration per session, or stage II or III COPD [135]. Only in patients with IMT were positive adaptations on performance and pulmonary parameters evident [135].

Another recent review of IMT and COPD evaluated 16 studies. This review also highlighted the heterogeneity of IMT protocols. The duration of IMT ranged from 3 weeks to 15 months, with sessions lasting from 3 to 60 minutes, 3 to 7 times weekly; the target pressure during inspiration was not well specified [136]. It seems that an IMT of less than 20 minutes is more effective for improving strength, as is a PImax less than 60%, while fewer than 3 sessions per week seem to yield little effect on diaphragmatic function [136].

A recent systematic review and meta-analysis, with GOLD stage II-IV COPD, with IMT added to PR in COPD, evaluated 9 studies, including 582 patients [137]. Rehabilitation duration ranged from 4 to 24 weeks, with sessions ranging from 2 per week to daily, with 1-2 sessions per day lasting 5-30 minutes each. The authors concluded that there was little evidence supporting the usefulness of adding IMT to PR to achieve greater rehabilitation benefits, and that the training protocols were heterogeneous.

Recent research suggests using glycemic thresholds to determine the appropriate amount of IMT in stable COPD patients, as the point of respiratory muscle exhaustion coincides with low glycemic levels; however, further studies and insights are lacking [138].

There is no gold standard for IMT. From this, we can deduce that we don't really know how to use IMT, and the uncertainty about whether this approach is unnecessary when added to PR stems from discrepancies in the literature rather than from the training itself.

ATS/ERS cites the American College of Sports Medicine (ACSM) to identify the appropriate program to follow based on the muscle metabolism modality of the limbs, such as aerobic and anaerobic activities [15]. To involve the white fibers (hypertrophy and strength), ACSM recommends using a 2-3-minute rest between one set and the next, using a resistance to overcome that corresponds to 60%-80% of the individual's one-repetition maximal (1RM) effort, with 8-12 repetitions; only one exercise per muscle group is sufficient, with a maximum of 4 sets [139]. To train the aerobic or endurance aspect, ACSM recommends using a maximum load of 50% of 1RM, 15-25 repetitions, and for a maximum of two sets. ACSM recommends waiting 48-72 hours before starting another training session during the week, with a maximum of 3 sessions per week, and one session that does not exceed 60 minutes. For exercise bike/treadmill training, the latest GOLD report recommends a fatigue work rate of around 60-80% of the heart rate derived from the cardiopulmonary exercise test, and/or a Borg value related to dyspnea and fatigue of 4-6 (moderate-severe level) [24].

The goal of IMT is not only to change or slow down the ultrastructural adaptations of the diaphragm, working with specific training regimens, but also to increase its range of motion (DEmax), which correlates with pulmonary values and symptom severity [22,120]. DEmax is a parameter for differentiating COPD from AECOPD, where the excursion is further reduced during the exacerbation period [140]. A low DEmax value coincides with weakness of the inspiratory accessory muscles and reduced performance capacity (Tables 1-2) [141]. We did not report effect sizes (e.g., mean differences) and their 95% confidence intervals (95% CIs), as our aim was not to conduct a systematic review of statistical significance.

**Table 1:** PR + IMT, versus PR alone. There appears to be no evidence that adding IMT to PR results in improved results. Various studies highlight the heterogeneity of results and work parameters. We did not report effect sizes (e.g., mean differences) and their 95% confidence intervals (95% CIs), as our aim was not to conduct a systematic review of statistical significance. PR: pulmonary rehabilitation; IMT: inspiratory muscle training; FEV1: forced expiratory volume in the first second; PImax: maximal inspiratory pressures; 6MWD-6MWT: six-minute walk distance-six-minute walk test.

Author, reference	Patient typology	Number of studies	Number of patients	Outcomes	Certainty of the evidence (GRADE) as an improvement in outcomes and/or statistical significance	Training	Duration of the study
Ammous et al. [9]	stable COPD	22	1446	Dyspnea	Moderate-certainty evidence/very low-certainty evidence, with no or low statistical significance	The applied resistance ranges from a minimum of 9% of PImax to 133%; an IMT session can last from 5 to 60 minutes, with a number of breaths against resistance ranging from 10 to 36, and with 2-15 sets; the increase in intensity for the patient ranges from 5% to 30% more than the initial load, with a variable time span between one increase and the next; the pauses between one set and the next, described only in rare cases, vary from 5 seconds to 10 minutes, for 4-5 minutes of duration for each set. Often, studies have been performed at home for IMT, with variable supervision	The days in which IMT is performed range from two days a week to every day, with a minimum of two weeks to 24 weeks
				6-minute walk distance (6MWD)	moderate-certainty evidence/very low-certainty evidence, with no or low statistical significance		
				Inspiratory muscle strength (inspiratory muscle strength (PImax)	moderate-certainty evidence/very low-certainty evidence, with no or low statistical significance		
				Health-related quality of life	Moderate-certainty evidence/very low-certainty evidence, with no or low statistical significance		
Dong et al. [38]	stable COPD	1	38	maximum diaphragmatic excursion (DEmax)	Statistically significant	30 breaths for each set, for two sets; from a PImax of 30% until reaching a resistance of 50% of the PImax. Sessions supervised by physiotherapists (once every 2 weeks)	24 weeks at home (12 weeks PR and then 12 weeks IMT) versus PR alone
				PImax	Statistically significant		
				6MWD	Statistically not significant		
				Dyspnea	Statistically significant		
Ellefsen et al. [46]	stable COPD	1	60 (male patients)	Dyspnea	Statistically significant	30 breaths per session, three times weekly (IMT; abdominal electrical stimulation. No PR was performed)	8 weeks
				6MWT			
				Severity of COPD: Evaluated using the COPD Assessment Test (CAT)	Statistically significant		
				Diaphragmatic thickness (DT)	Statistically significant		
Jiang et al. [47]	stable COPD	16	1364	Dyspnea	Statistically significant	IMT only: 3 minutes to 60 minutes, 3 times a week up to 7 times weekly; <60%- ≥60% PImax	From 3 weeks to 15 months
				PImax	Statistically significant		
				Quality of life (QOL)	Statistically significant		
Fard et al. [48]	stable COPD	9	582	QOL	Low-quality evidence	IMT and PR, versus PR alone. Sessions ranging from 2 per week to daily, with 1-2 sessions per day lasting 5-30 minutes each	From 4 to 24 weeks
				6MWD	Low-quality evidence		
				PImax	Low-quality evidence		
				FEV1, FEV1%	Low-quality evidence		

**Table 2:** The table summarizes the indications that the major scientific societies and organizations have for IMT and COPD IMT: inspiratory muscle training; AECOPD: acute exacerbation of chronic obstructive pulmonary disease; COPD: chronic obstructive pulmonary disease; PImax: maximal inspiratory pressures; GOLD: Global Initiative for Chronic Obstructive Lung Disease

Society/organization	Type of patients	IMT parameters	References
American Thoracic Society/European Respiratory Society	Stable COPD, AECOPD no indication	PImax equal to or exceeding 30%	[15,16]
British Thoracic Society	Stable COPD	No indication	[17]
2025 GOLD Report	No indication	No indication	[24]

Changing IMT parameters

Based on available literature, the COPD diaphragm is weak and has a lower capacity to withstand demanding workloads due to a systemic inflammatory environment, which makes the inspiratory muscle less capable of repairing itself, prolonging recovery times [22,142]. In healthy subjects, the diaphragm begins to fatigue at a PImax of 50%, and it takes 30 minutes for the muscle to fully recover (as measured by electromyography) [143]. It would likely be best not to exceed the 50% PImax threshold to avoid further damage to the muscle [22].

We do not know the diaphragm muscle recovery time in COPD patients after IMT between sessions during the week, nor how long to wait before repeating a series of resisted breaths. Probably, recovery times should be spread out within a training session. We don't know the most useful percentage increase to improve performance between sessions.

We could hypothesize that 50% of PImax in COPD patients is an anaerobic activity and represents the work threshold indicated by ACSM (60-80% of 1RM), while a lower threshold, such as 15% of PImax, is purely aerobic work and reflects the values dictated by ACSM (50% of 1RM). Low-resistance loads in IMT have already been recommended by other authors [22].

We must also consider that when the patient performs PR, the diaphragm is always involved, and if IMT is added, it must be kept in mind that the inspiratory muscle must recover from previous training [22]. The inclusion of IMT must also be chosen considering the aerobic/anaerobic activity within PR, without overworking the diaphragm.

An IMT aimed at improving aerobic metabolism should not exceed 20% of PImax, with 15-25 breaths, for two sets, and with a several-minute rest. An IMT aimed at improving anaerobic metabolism should not exceed 50% of PImax, 8-12 breaths, with a 30-minute rest between sets, and probably a maximum of two sets (to keep the workout under 60 minutes). Each IMT session should be scheduled every 3 days to comply with ACSM recommendations; furthermore, the two IMT modalities should not be performed on the same day but separated into different weekly sessions, and not during physical activities such as cycling/weight training. Each breath, regardless of the percentage of inspiratory resistance, should be deep but without involving shoulder shrugs. The patient should be instructed that, during IMT, other accessory inspiratory muscles should not be recruited to compensate for diaphragmatic weakness or incoordination. To do this, the patient may wish to observe IMT in front of a mirror.

To increase training load (PImax percentage), when the patient reports a lower Borg scale value, and the number of breaths tends to increase despite the same resistance, the clinician may increase the PImax resistance percentage. This allows for a truly subjective assessment of training load. If the patient shows a decline in diaphragmatic performance, the training load (duration, load, sessions, sets, and repetitions) should be decreased or stopped (if there is an exacerbation). A decline in performance could mean that the patient is undergoing an overtraining stimulus compared to their recovery capacity.

IMT should be performed throughout the patient's life, as pathological conditions do not improve, and the diaphragm will always need the most physiological stimuli possible. Research should further explore and use these parameters to determine whether IMT can offer more clinically relevant results. The review highlighted that there is no gold standard for IMT, despite the diaphragm muscle's great importance.

Take-home message: Currently, we don't know the best parameters for organizing IMT.

Other gray areas

We've highlighted the inconsistency of PR/IMT parameters used in experimental research. Many other questions need to be answered.

We don't know whether patients work in groups or individually during training in a hospital setting: is one more effective than the other? We don't know. Similarly, we don't know whether telemedicine delivered via a group video call or with a single patient is better.

When organizing and planning an experimental study, the clinician and/or statistician must create groups of patients that are homogeneous and easily comparable in terms of data and values. Most of the time, comorbidities are not taken into consideration, which alters homogeneity but eliminates clinical reality [53]. Patients with COPD suffer from many comorbidities and concomitant dysfunctions, highlighting the different endotypes/phenotypes. We can find cardiovascular pathologies (chronic heart failure, arrhythmias, hypertension, peripheral arterial disease, cerebrovascular disease), mood disorders (anxiety, depression, stress), sinusitis/rhinosinusitis, OSA, diabetes, dyslipidemia, periodontal disease, obesity, sarcopenia, deficiency anemias, renal failure, hypothyroidism, neurological disease, osteoporosis, gastroesophageal reflux, lung cancer, noncirrhotic liver disease, bronchiectasis [24,68,127,144-148]. Furthermore, some studies do not take into consideration smokers compared to non-smokers. For example, smokers, before the detection of COPD, already present ultrastructural alterations of the diaphragm [149]. Patients with COPD may present dyspnea, productive cough, frequent exacerbations, and not all these conditions are always concomitant in different patients; there may be genetic predispositions, such as alpha-1 antitrypsin deficiency, telomere polymorphisms [149]. Not all patients defined as eosinophilic COPD respond equally to the same therapy [149]. We do not know which training indications, depending on the phenotype/endotype, can give a greater clinical response.

There are differences between females and males. Women show greater dyspnea, and under 65 years of age, they record a higher number of exacerbations than males, despite exhibiting lower rates of smoking; women suffer more alterations in the psychological/psychiatric sphere [150,151]. Men present a higher percentage of cardiovascular pathologies, as well as a higher average FEV1 value than women; females are more likely to use non-invasive ventilation during hospitalization [11]. We do not know whether IMT should have different indications depending on gender.

Some patients may present with respiratory symptoms or structural airway alterations, but without obstruction. This type of patient is considered pre-COPD and classified as PRISm (preserved ratio impaired bronchodilation) [24]. These patients may develop COPD, but not all PRISm patients develop irreversible obstruction, regardless of smoking or comorbidities [126,127]. We have no data on how to set PR/IMT with these patients.

There is a difference between the patient's age and COPD diagnosis. Under 50 years of age, a COPD diagnosis appears to predict premature mortality and fatal cardiac events [152]. Should we change the training setting? We have no data.

Another potential source of discrepancy in the results of different studies is the fact that clinicians writing the article often don't follow patients during PR/IMT; the clinician simply analyzes the data, which may have been entered by a statistician or specialist in a specific file, derived from the medical record, and chooses which data to analyze. Therefore, the data constrained by this choice are considered to ensure consistency in the published text; however, this negatively impacts clinical reasoning by eliminating subjectivity. In the current state of evidence, there are further limitations, including the small sample sizes in many studies and the considerable variability in reported outcomes. Research should investigate the applicability of the training protocol to routine respiratory physiotherapy in health services; build prospects for multicentre studies with larger samples and long-term follow-up; and explore incorporating training as a complementary strategy in pulmonary rehabilitation programs, with cost-effectiveness analysis. We would like to remind you that this article is a narrative review (non-systematic method, single database, no formal RoB), and further studies will need to be carried out to avoid errors.

Take-home message: The literature should make further efforts to eliminate the multiple discrepancies in results in studies using IMT/PR.

## Conclusions

COPD is a respiratory disease that causes progressive airway obstruction, representing the third leading cause of mortality worldwide. One of the treatments available is PR, which involves aerobic and anaerobic physical activity and, not always, specific IMT. The article reviewed the importance of IMT and its associated positive effects, as well as the physiological and pathological adaptations of the diaphragm, highlighting discrepancies among protocols reported in the literature. The article posited a new hypothesis regarding workloads for the inspiratory muscles, both aerobic and anaerobic, while attempting to respect current knowledge of training principles. Further research should focus on better understanding the most effective parameters for helping patients with IMT and PR, also considering phenotypic/endotypic differences.
